# Association of routine hematological parameters with the development of monoclonal gammopathies: a case-control study of 134,740 patients: **Resubmitted to annals of Hematology 26 March 2024**

**DOI:** 10.1007/s00277-024-05822-9

**Published:** 2024-06-06

**Authors:** Jakob Røllum-Larsen, Anna Elise Engell, Marta Diaz-delCastillo, Anne-Marie Heegaard, Henrik Løvendahl Jørgensen

**Affiliations:** 1 Department of Clinical Biochemistry, Copenhagen University Hospital Hvidovre, Hvidovre, Denmark; 2 Department of Forensic Medicine, University of Aarhus, Aarhus, Denmark; 3 Danish Spatial Imaging Consortium (DanSIC), Aarhus, Denmark; 4 Department of Drug Design and Pharmacology, University of Copenhagen, Universitetsparken 2, Copenhagen, 2100 Denmark; 5 Department of Clinical Medicine, University of Copenhagen, Blegdamsvej 3B, 2200 KBH N, Copenhagen, Denmark

**Keywords:** Multiple myeloma, Paraproteinemia, Cancer development, Complete blood count, Retrospective study

## Abstract

The diagnosis of multiple myeloma requires detection of paraproteinemia and confirmation of monoclonal bone marrow infiltration, along with signs of end-organ damage. Despite the increasing prevalence, serum paraproteinemia is not routinely measured. We examined the relationship between alterations in routine hematological parameters and the development of paraproteinemia in a case-control study. Data was retrieved from a laboratory database in the capital region of Denmark between 01/01/2012 and 31/12/2022. Patients were included if they had a test for paraproteinemia (*n* = 134,740) and at least one prior hematological parameter (white blood cells, hemoglobin and platelet count) with a minimum follow-up of 1 year.

Between 96,999 and 103,590 patients were included in each of the three hematological groups. We found white blood cell count and the presence of paraproteinemia followed an inverse J-shaped curve, with the highest presence below 3 × 10^9^/L and above > 9 × 10^9^/L. The adjusted OR below and above the nadir of 4 × 10^9^/L was 1.61 (95% CI 1.25; 2.08, *p* < 0.0001) and 1.03 (95% CI 1.03; 1.04, *p* < 0.0001). Hemoglobin levels were inversely associated the presence of paraproteinemia, with the highest association below 6 mmol/L with an OR of 1.30 (95% CI 1.28; 1.32, *p* < 0.0001) adjusted for age and gender. Platelet count followed a U-shaped curve with the highest association at < 100 × 10^9^/L. The adjusted OR below and above the nadir of 250 × 10^9^/L was 1.13 (95% CI 1.10; 1.17, *p* < 0.0001) and 1.10 (95% CI 1.08; 1.12, *p* < 0.0001) respectively. In conclusion, all three parameters showed significant association with later paraproteinemia.

**Supplementary Information:**

The online version contains supplementary material available at 10.1007/s00277-024-05822-9.

## Introduction

Multiple Myeloma (MM) is a malignant hematological cancer characterized by abnormal plasma cell proliferation in the bone marrow [1]. The global incidence of MM is rising with a current incidence of 2.1/100,000 and 1.4/100,000 for men and women respectively, and even higher regional incidences in Australia, Western Europe and United States of America [2]. In total, MM constitutes 10% of all hematological malignancies and 1% of all cancers [3, 4]. The diagnosis of overt MM requires the presence of ≥ 10% clonal plasma cells in the bone marrow and the development of at least one myeloma defining event, such as CRAB criteria (calcium elevation, renal insufficiency, anemia, bone disease) or one or more myeloma-defining biomarkers: ≥60% clonal plasma cells in the bone marrow, a serum light free chain (FLC) ratio of ≥ 100 in combination with a concentration of FLC above 10 mg/dL or > 1 focal lesions (> 5 mm) on MRI [5, 6].

MM is typically preceded by one or both of its two asymptomatic precursor conditions: Monoclonal Gammopathy of Undetermined Significance (MGUS) or Smoldering Multiple Myeloma (SMM) [7]. The defining diagnostic criteria between MGUS and SMM is based on the concentration of serum paraprotein and monoclonal plasma cells in the bone marrow; MGUS is characterized by less than 10% monoclonal plasma cells in the bone marrow, while more than 10% are present in SMM [7, 8]. MGUS prevalence is up to 5.3% in the general population over 70 years old [9], and presents a constant annual risk of progression to MM of 1%, which increases to a cumulative 10% in SMM [5, 8, 10].

Despite MM being a life-threatening condition with a 5 year survival of 53.9% in the US and 62.4% in Germany, the prognosis and overall survival have improved remarkably over the last decades [2, 11]. Importantly, early detection of premalignant gammopathies has been correlated with increased overall survival in MM through increased monitoring and timely treatment [12, 13]. Nonetheless, current MGUS and SMM diagnosis remain incidental and specific paraprotein measures are not routinely conducted as part of the standard of care hematological investigations. In order to accurately predict survival and prognosis of various cancer types, the Hemoglobin, Albumin, Lymphocytes and Platelets Score (HALP) has been effectively used [14]. In MM, the HALP score has been proven as a prognostic marker for overall and progression-free survival in MM [4], but the association between routinely measured serum biomarkers and premalignant gammopathies remains unknown. Therefore, the aim of this retrospective case-control study is to examine the relationship between alterations in blood hemoglobin, white blood cell and platelet count, and the development of paraproteinemia.

## Methods

### Patient population and data collection

This study includes data from patients who had a diagnostic blood sample taken for potential monoclonal gammopathies, identified using the NPU17675 code for Plasma M protein, the NPU19606 code for Kappa light chain monoclonal gammopathy or the NPU19607 code for Lambda light chain monoclonal gammopathy, between 01/01/2012 and 31/12/2022. Patients were referred to the analysis according to national guidelines [15]. Inclusion criteria also required data availability for prior hematological parameters, including hemoglobin, white blood cells and/or platelet count. The data was extracted from LAKBA II, a database containing blood test results from patients in hospitals and general practices in the Capital Region of Denmark.

Only patients with a minimum follow-up of 1 year between hematological parameters and M protein/free light chain testing were included. If a patient had multiple hematological assessments during the 11-year study period, their mean was used in the further analyses. If a patient had a positive M protein or free light chain test result, this result was used in the analyses. Otherwise, if multiple negative test results were present, only the first was included in this study.

A total of 134,740 patients who had a test for monoclonal gammopathy were identified. In this group, 133,792 patients also had hemoglobin values available. Likewise, 133,612 patients had available white blood cell data, and 133,303 had platelet count data. Patients with a test for monoclonal gammopathy taken before the respective hematological parameters were excluded. Further information can be found in the supplementary Figs. 1–3.

### Measurements of hematological parameters, M protein and free light chains

The analysis of the hemoglobin, white blood cell and platelet count was performed by all laboratories located in the capital region of Denmark. The analyses of M protein (NPU17675), Kappa light chain monoclonal gammopathies (NPU19606) and Lambda light chain monoclonal gammopathies (NPU19607) were performed by four specialized laboratories in the region. The majority of the M-protein samples, 94.4%, were analyzed by capillary electrophoresis using the Capillarys platform from Sebia (Lisses, France). The remaining 5.6% were analyzed using agarose gel electrophoresis. According to the national guideline [15], when the M-protein analysis (NPU17675) was negative, the samples were screened for light chain monoclonal gammopathies. In the included laboratories, this was performed on the Atellica platform from Siemens Healthineers (Erlangen, Germany), on the SPAPLUS platform from Binding Site or on the Optilite platform from Binding Site.

The test was considered positive for Kappa light chain monoclonal gammopathy at concentrations above 22.4 mg/L. For Lambda light chain monoclonal gammopathy, the cut-off used was 27.0 mg/L.

All included laboratories participated in external quality programs during the study period to confirm the reliability of the assays over time.

### Statistical analyses

Differences between continuous variables were tested using unpaired t-tests or Mann Whitney U tests as appropriate while differences in the distribution of categorical variables were tested using Chi-square tests.

In order to investigate the shape of the association between the hematological parameters and the development of monoclonal gammopathy, we plotted rounded values of the mean concentration of white blood cells, hemoglobin and platelets on the x-axis versus the percentage of patients with monoclonal gammopathy on the Y-axis. For white blood cells and platelets, the association was approximately inverse J-shaped and U-shaped, respectively. The nadir of the two curves was therefore used to split the data into below and above this value for further analyses.

Unadjusted and adjusted for covariates (i.e. age and gender) proportional hazards regression analyses were used to investigate the relationship between monoclonal gammopathy and the three hematological parameters.

All analyses were performed using SAS version 9.4 (SAS Institute, Cary, NC, USA). For all the statistical analyses, *p* < 0.05 was considered statistically significant.

### Ethics

The data from the laboratory information system was anonymized before analysis. In Denmark, no approval from the Ethics Committee is required for this type of register-based study.

## Results

A total of 99,766 patients with an M protein/free light chain test result and at least one white blood cell count in the study period between 01/01/2012 and 31/12/2022 were included. Likewise, 103,590 with a hemoglobin sample and M protein/free light chain, and 96,999 patients with a platelet count and M protein/free light chain were included.

Table 1 shows the basic characteristics of the study population according to the hematological and monoclonal gammopathy status. In all the three groups with monoclonal gammopathy, the mean age at the time of the first hematological measurement was significantly higher, compared with the groups without monoclonal gammopathy. The follow-up period between the measurements of the hematological parameters and the M protein/free light chain test was significantly longer in all of the groups with monoclonal gammopathy. Concerning the gender distribution, paraproteinemia was more prevalent in males than females.

**Table 1:** Monoclonal gammopathies is defined as having a positive plasma M protein, Kappa light chain or Lambda light chain test Age at first hematologic measurement, white blood cell, hemoglobin, platelets: Mean (SD) Follow-up: median (IQR) of all hematologic measurements. Gender: N (%)

	Monoclonal gammopathy	No monoclonal gammopathy
**White blood cells (10^9/L)**	8.8 (8.6)	8.3 (5.7)
Number of white blood cell measurements	571,253	778,786
Number of unique patients	29,760	70,006
Age (years)	65.0 (12.6)	55.7 (16.7)
Follow-up (years)	4.1 (2.3; 6.6)	3.8 (2.2; 6.2)
Gender (males/females)	15,434 (52) / 14,326 (48)	26,671 (38) / 43,335 (62)
**Hemoglobin (mmol/L)**	7.7(1.3)	8.0 (1.2)
Number of hemoglobin measurements	708,902	929,995
Number of unique patients	30,610	72,980
Age (years)	64.6 (12.5)	55.4 (16.7)
Follow-up (years)	4.0 (2.3; 6.5)	3.8 (2.2; 6.1)
Gender (males/females)	15,939 (52) / 14,671 (48)	28,002 (38) / 44,978 (62)
**Platelets (10^9/L)**	271 (124)	271 (111)
Number of platelet measurements	136,356	951,802
Number of unique patients	29,255	67,744
Age (years)	65.2 (12.6)	56.0 (12.6)
Follow-up (years)	4.0 (2.3; 6.5)	3.8 (2.2; 6.1)
Gender (males/females)	15,163 (52) / 14,092 (48)	25,707 (38) / 42,037 (62)

**Fig. 1:**
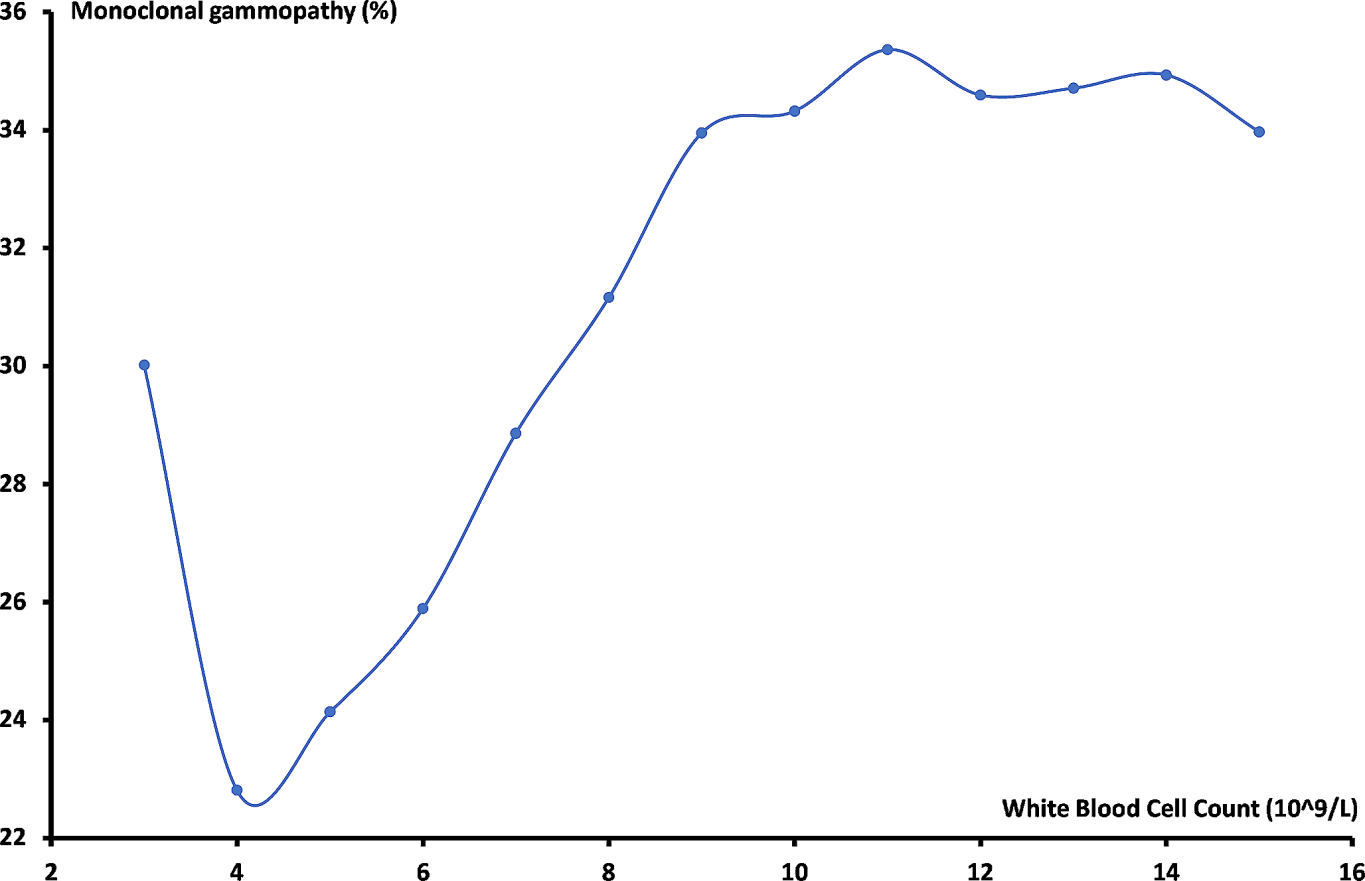
The association between white blood cell count and the percentage of patients with a positive M-protein/FLC test

The association between white blood cell count and the presence of monoclonal gammopathy during the follow-up period is illustrated in Fig. 1. The curve is reversed J-shaped with increasing presence (up to 30%) of monoclonal gammopathy below a mean white blood cell count of 4 × 10^9^/L and above a mean count of 12 × 10^9^/L the presence of monoclonal gammopathy was 35%.

**Fig. 2:**
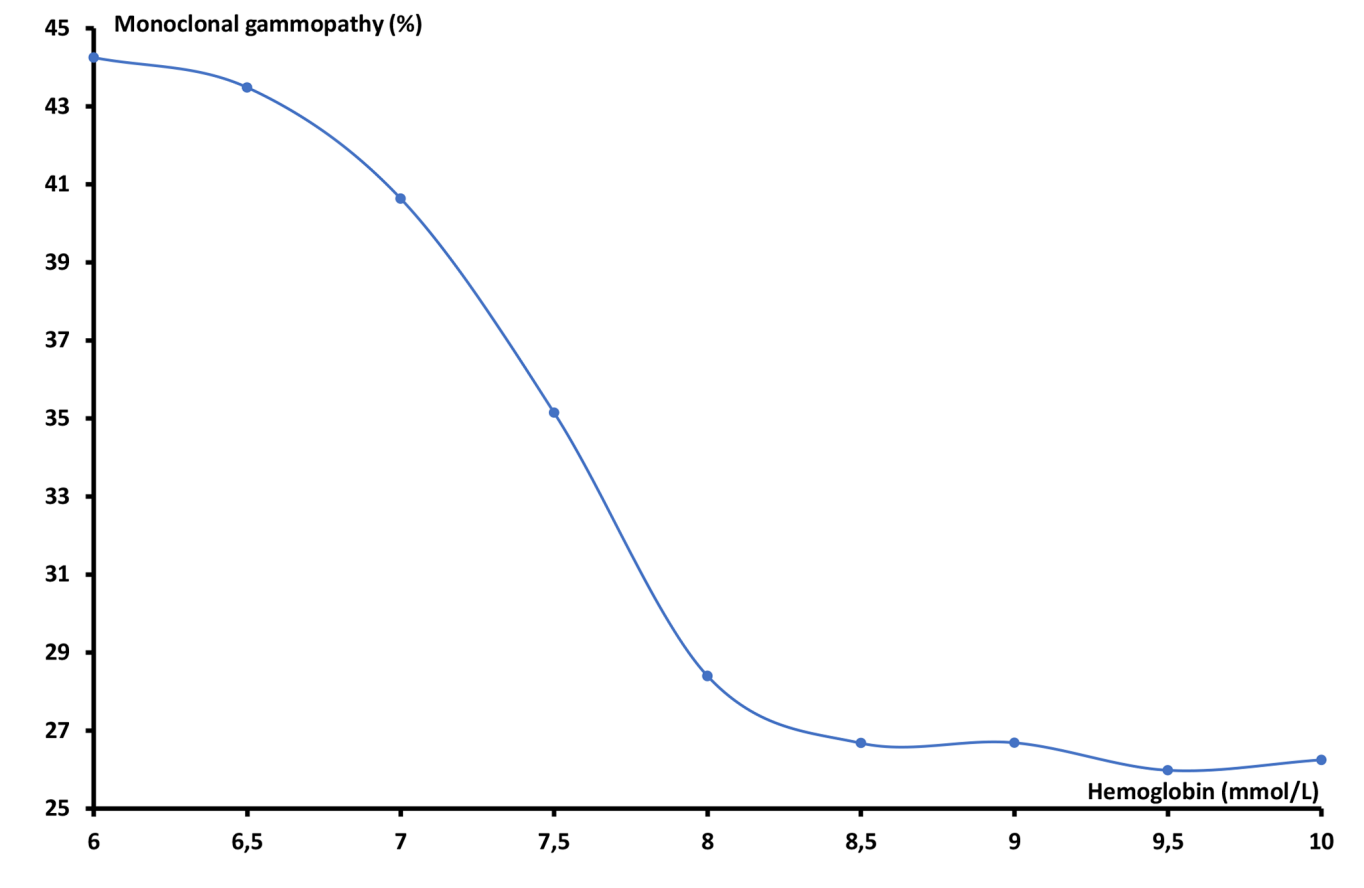
The association between hemoglobin level and the percentage of patients with a positive M-protein/FLC test

For hemoglobin, the presence of monoclonal gammopathy was inversely correlated with blood hemoglobin levels (Fig. 2). The presence of monoclonal gammopathy was highest (44%) at hemoglobin levels below 6 mmol/L, and decreased to a minimum of 26% correlating to hemoglobin levels between 8.5 and 10 mmol/L.

**Fig. 3:**
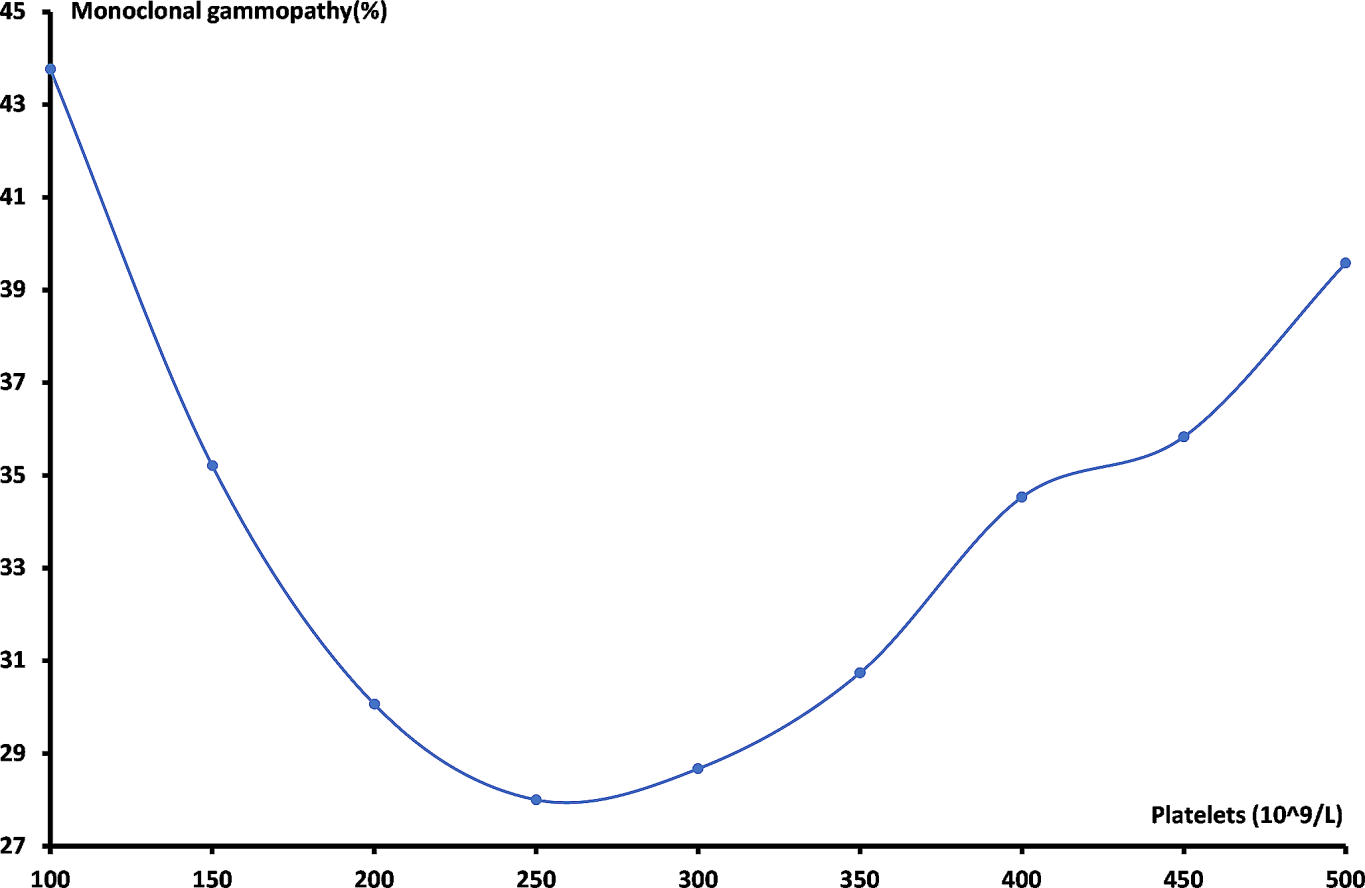
The association between platelet count and the percentage of patients with a positive M-protein/FLC test

Lastly, the association between platelet count and the presence of monoclonal gammopathy followed a U-shape (Fig. 3), with the highest presence (> 40%) at platelet counts < 100 × 10^9^/L. The presence of monoclonal gammopathy decreased with increasing platelet counts, with nadir at 250 × 10^9^/L correlating to a presence of monoclonal gammopathy of 28%. After the nadir, the presence increased towards > 500 × 10^9^/L.

The unadjusted Odds Ratios (OR) for the three hematological parameters as well as the ORs of each parameter adjusted for gender and age were also calculated (Table 2). The white blood cell count followed a reversed J-shaped curve, and therefore separate OR were calculated for each side of the nadir at 4 × 10^9^/L. Below the nadir, the unadjusted OR was found to be 1.68 (95% CI 1.32; 2.14, *p* < 0.0001) and above the nadir the OR was 1.04 (95% CI 1.04; 1.05 *p* < 0.0001). When adjusted for age and gender, the ORs for the descending and ascending parts of the curve were 1.61 (95% CI 1.25; 2.08, *p* < 0.0001) and 1.03 (95% CI 1.03; 1.04, *p* < 0.0001), respectively. The relation between hemoglobin levels and the presence of monoclonal gammopathy showed a linearly descending curve, with an unadjusted OR of 1.25 (95% CI 1.24; 1.27, *p* < 0.0001). When adjusted for gender and age the OR was 1.30 (95% CI 1.28; 1.32, *p* < 0.0001). Finally, the OR for platelet count was calculated and as for the association between white blood cells and the presence of monoclonal gammopathy, the curve was split and two separate ORs were calculated as the curve followed a U-shape. The unadjusted OR of the descending part below the nadir of 250 × 10^9^/L was 1.26 (95% CI 1.22; 1.29, *p* < 0.0001) and the unadjusted OR above the nadir was 1.10 (95% CI 1.09; 1.12, *p* < 0.0001). Similarly, the ORs for platelets were adjusted for gender and age, resulting in OR 1.13 (95% CI 1.10; 1.17, *p* < 0.0001) below the nadir and 1.10 (95% CI 1.08; 1.12, *p* < 0.0001) above.

Additionally, the white blood cell/platelet count ratio was estimated, but did not show greater predictive value compared to the three hematological parameters separately, and was therefore not included in further analyses (data not shown).

**Table 2:** Unadjusted and adjusted odds ratios for having monoclonal gammopathy for each of the hematological parameters

	Unadjusted	*P*-value	Adjusted	*P*-value
**Leucocytes < 4 x 10^9/L**				
Leucocytes (per 10^9/L decrease)	1.68 (1.32; 2.14)	< 0.0001	1.61 (1.25; 2.08)	< 0.0001
Age (per 5 yrs)	-	-	1.25 (1.20; 1.30)	< 0.0001
Male versus female	-	-	1.46 (1.15; 1.85)	< 0.0001
**Leucocytes > = 4 x 10^9/L**				
Leucocytes (per 10^9/L)	1.04 (1.04; 1.05)	< 0.0001	1.03 (1.03; 1.04)	< 0.0001
Age (per 5 yrs)	-	-	1.23 (1.22; 1.24)	< 0.0001
Male versus female	-	-	1.73 (1.68; 1.78)	< 0.0001
**Hemoglobin**				
Hemoglobin (per mmol/L decrease)	1.25 (1.24; 1.27)	< 0.0001	1.30 (1.28; 1.32)	< 0.0001
Age (per 5 yrs)	-	-	1.21 (1.21; 1.22)	< 0.0001
Male versus female	-	-	2.02 (1.96; 2.08)	< 0.0001
**Thrombocytes < 250 x 10^9**				
Thrombocytes (per 50 x 10^9/L decrease)	1.26 (1.22; 1.29)	< 0.0001	1.13 (1.10; 1.17)	< 0.0001
Age (per 5 yrs)	-	-	1.23 (1.22; 1.24)	< 0.0001
Male versus female	-	-	1.70 (1.63; 1.77)	< 0.0001
**Thrombocytes > = 250 x 10^9**				
Thrombocytes (per 50 x 10^9/L)	1.10 (1.09; 1.12)	< 0.0001	1.10 (1.08; 1.12)	< 0.0001
Age (per 5 yrs)	-	-	1.23 (1.22; 1.24)	< 0.0001
Male versus female	-	-	1.87 (1.78; 1.95)	< 0.0001

## Discussion

MM remains an incurable disease, but early detection and monitoring of its premalignant precursors have been associated with greater overall survival due to timely treatment intervention [12]. In this retrospective case-control study, we found that the results of routine hematological parameters may warrant further evaluation of potential paraproteinemia utmost importance to improve early detection and diagnosis. The goal of this study was to evaluate the association between blood hemoglobin, platelet and white blood cell counts and serum paraproteinemia in retrospective Danish data collected between 2012 and 2022. Our study found a significant relationship between hematological parameters and the presence of monoclonal gammopathy. Male gender led to a significant increase in paraproteinemia OR compared to female gender, and age was found to increase OR in all the analyses.

The relationship between hematological parameters and the later development of MGUS or MM has previously been investigated by Li et al. [16]. In a case-control study, they examined the whole blood count of 21 MM patients and 21 healthy controls at time of enrollment in the study. They found a significant reduction in both hemoglobin, platelet count and white blood cell count in MM patients. This data is in line with our findings of increased risk for monoclonal gammopathy development at low hemoglobin levels and low platelet counts, and also a greater OR at low white blood cell count compared to higher levels.

An observational cohort-study of Danish patients without prior cancer diagnosis by Boennelykke et al. [17] sought to determine the effect of new-onset anemia on predicting a later cancer development within a follow-up of 12 months. The study found a significant relationship between all types of anemias and the incidence ratio of cancer, primarily of the gastrointestinal or respiratory systems. Among patients with unclassified anemia, hematological cancers had the highest standardized incidence ratio between both genders. The study further found that risk of cancer development increased continuously with age and also with the severity of anemia. This relationship between degree of anemia and risk of cancer development follows the same relationship as found between hemoglobin concentration and risk of monoclonal gammopathy in our study.

White blood cell count has been investigated in several studies as a prognostic biomarker for the development of various cancers. A study by Margolis et al. [18] investigated the incidence and mortality of breast, colorectal, endometrial and lung cancer in a prospective cohort with baseline measures of several hematological parameters collected at study initiation, including white blood cell counts. The outcomes were the incidence of cancer and cancer mortality. The study found that with increasing white blood cell count, the incidence of all four cancers also rose, suggesting a significant association between cancer development and systemic immunoreactivity. A study on the relationship between white blood cells and cancer incidence was conducted by Wong et al. [19], where data from patients in the UK biobank database was analyzed. The study found that increasing white blood cell count was associated with an increase in the incidence of lung cancer with an average follow-up of 7 years. While both of these studies showed a rising incidence in cancer development with increased white blood cell count, our study found a reversed J-shape relationship between white blood cell count and serum paraproteinemia. Importantly, both of the aforementioned studies used white blood cell data divided in quartiles, compared to our study in which white blood cell count was analyzed as a continuous variable, which may explain the differences across studies. Moreover, our study focused on patients with potential hematological cancer development, which may affect hematological parameters more acutely than solid cancers contained in organs compared to cancers in the bone marrow. Nonetheless rising white blood cell count has been shown to correlate to the development of several cancers.

Finally, the relationship between cancer incidence and platelet count has been examined in a study Jensvoll et al. [20]. The study examined the risk of developing cancer and the risk of venous thromboembolisms in a Norwegian prospective population-study with cancer-free participants, and found no relation between platelet count and the risk of developing cancer. This is in contrast with our results, where thrombocytes and monoclonal gammopathy presented an U-shape association.

Several studies have examined the importance of the combination of hemoglobin, lymphoctyte count, platelet count and albumin in the HALP score in relation to prognosis and severity of several cancers, including MM [4, 14, 21, 22]. The value of the HALP scores as a prognostic biomarker of MGUS or MM development has yet to be examined, but the data from our study showed that all three hematological parameters had a predictive value, and the addition of albumin and lymphocyte count to this might yield interesting results. Future studies will investigate whether the addition of albumin and lymphocyte data will clarify the prognostic value of HALP in the development of monoclonal gammopathies.

### Strengths and weaknesses

An important limitation of our study is that the population only included patients who in their lifetime had a blood test taken for M protein/free light chains; all patients without this test were excluded. Serum paraprotein is tested on suspected development of MGUS or MM, and these patients typically present themselves with symptoms including fatigue, bone pain or anemia, amongst others [5]. Asymptomatic patients are therefore more unlikely to have a test for M protein/free light chains taken, and this large group of patients are missing from the dataset of this study. This could potentially result in selection bias of the study population. Sigurbergsdóttir et al. thus found that patients with a clinical diagnosis of MGUS have a higher mean number of comorbidities and are more likely to have been diagnosed with certain medical conditions compared to a screening population [23]. Furthermore, the prevalence of MM differs greatly between races and countries [2]. This information is not incorporated in the patient population of our study, and therefore it is not possible to take that into account in our statistical models. Additionally, given the long-term course of monoclonal gammopathies, it is possible that MGUS or even MM was already present at the time of blood test of the hematological parameters. This study only describes the biochemical status in the years prior to the positive M-protein/FLC test and cannot, therefore, specify the predictive value of the investigated parameters.

Our study was designed to analyze the association between routine hematological parameters and the later presence of paraproteinemia development a large population of Danish patients. We found that decreased hemoglobin, platelet and white blood cell count are all associated with increased presence of paraproteinemia, suggesting that these routine biomarkers can be utilized to inform the early and easy screening of potential MGUS, SMM and MM patients. The Iceland Screens, Treats, or Prevents Multiple Myeloma (iStopMM) study is a nationwide ongoing screening program for multiple myeloma (MM) and its precursors including 75,422 participants [24]. Rögnvaldsson et al. seek to determine the effect of follow-up on the survival of MGUS-patients. This includes continuous blood sampling in the study period, and the results to come from this study might corroborate the results of our study on the importance of routine blood samples in hematological malignancies and gammopathies [24].

## Conclusion

In conclusion, our study found a relationship between blood levels of hemoglobin, white blood cell and platelet count and the later presence of paraproteinemia. All three parameters showed a higher association with the presence of monoclonal gammopathy at low levels. Furthermore, both platelet count and white blood cell count were similarly associated with a higher presence of monoclonal gammopathy at higher levels. Overall, our results suggest that alterations in these routine hematological parameters can be clinically used to inform serum M protein and FLC testing and may promote the early detection of abnormal plasma cell growth.

### Electronic supplementary material

Below is the link to the electronic supplementary material.

**Supplementary Fig. 1**: Flowchart of the in- and exclusion process of the study for patients with a white blood cell count (WBC).

**Supplementary Fig. 2**: Flowchart of the in- and exclusion process of the study for patients with a hemoglobin measurement.

**Supplementary Fig. 3**: Flowchart of the in- and exclusion process of the study for patients with a platelet count.

## Data Availability

Data is not publicly available.
