# Supplementary material for: Association of routine hematological parameters with the development of monoclonal gammopathies: a case-control study of 134,740 patients: Resubmitted to annals of Hematology 26 March 2024
Source: Ann Hematol. 2024 Jun 6;103(8):3005–13. doi: 10.1007/s00277-024-05822-9 (PMC11283380; doi:10.1007/s00277-024-05822-9)
Supplement: Supplementary file 1 — Supplementary Fig. 1: Flowchart of the in- and exclusion process of the study for patients with a white blood cell count (WBC). [file 277_2024_5822_MOESM1_ESM.pptx]

## Slide 1
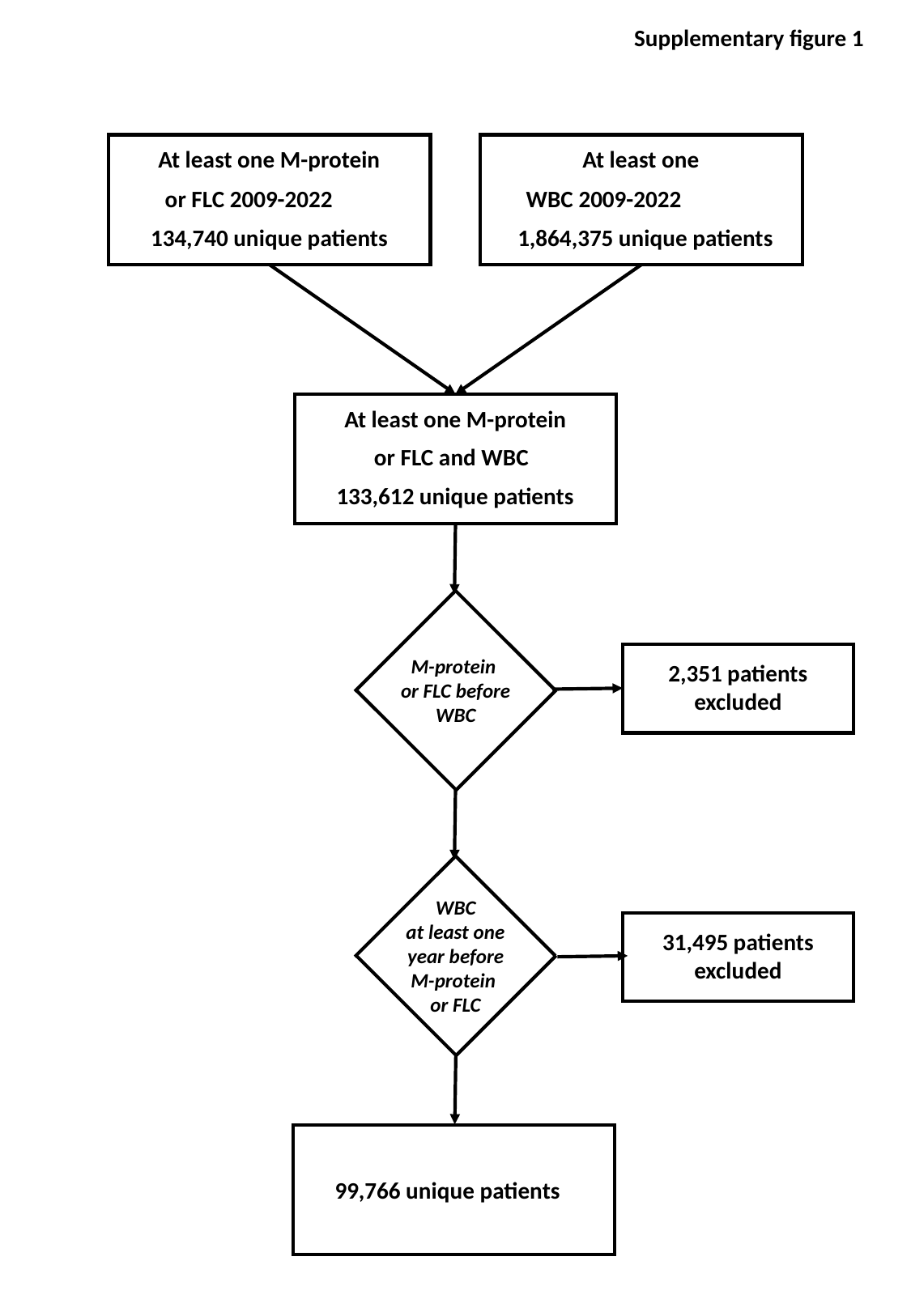

Supplementary figure 1
At least one M-protein
or FLC 2009-2022
134,740 unique patients
At least one
WBC 2009-2022
1,864,375 unique patients
At least one M-protein
or FLC and WBC
133,612 unique patients
M-protein
or FLC before
WBC
2,351 patients
excluded
WBC
at least one
year before
M-protein
or FLC
31,495 patients
excluded
99,766 unique patients
