# Supplementary material for: Association of routine hematological parameters with the development of monoclonal gammopathies: a case-control study of 134,740 patients: Resubmitted to annals of Hematology 26 March 2024
Source: Ann Hematol. 2024 Jun 6;103(8):3005–13. doi: 10.1007/s00277-024-05822-9 (PMC11283380; doi:10.1007/s00277-024-05822-9)
Supplement: Supplementary file 3 — Supplementary Fig. 3: Flowchart of the in- and exclusion process of the study for patients with a platelet count. [file 277_2024_5822_MOESM3_ESM.pptx]

## Slide 1
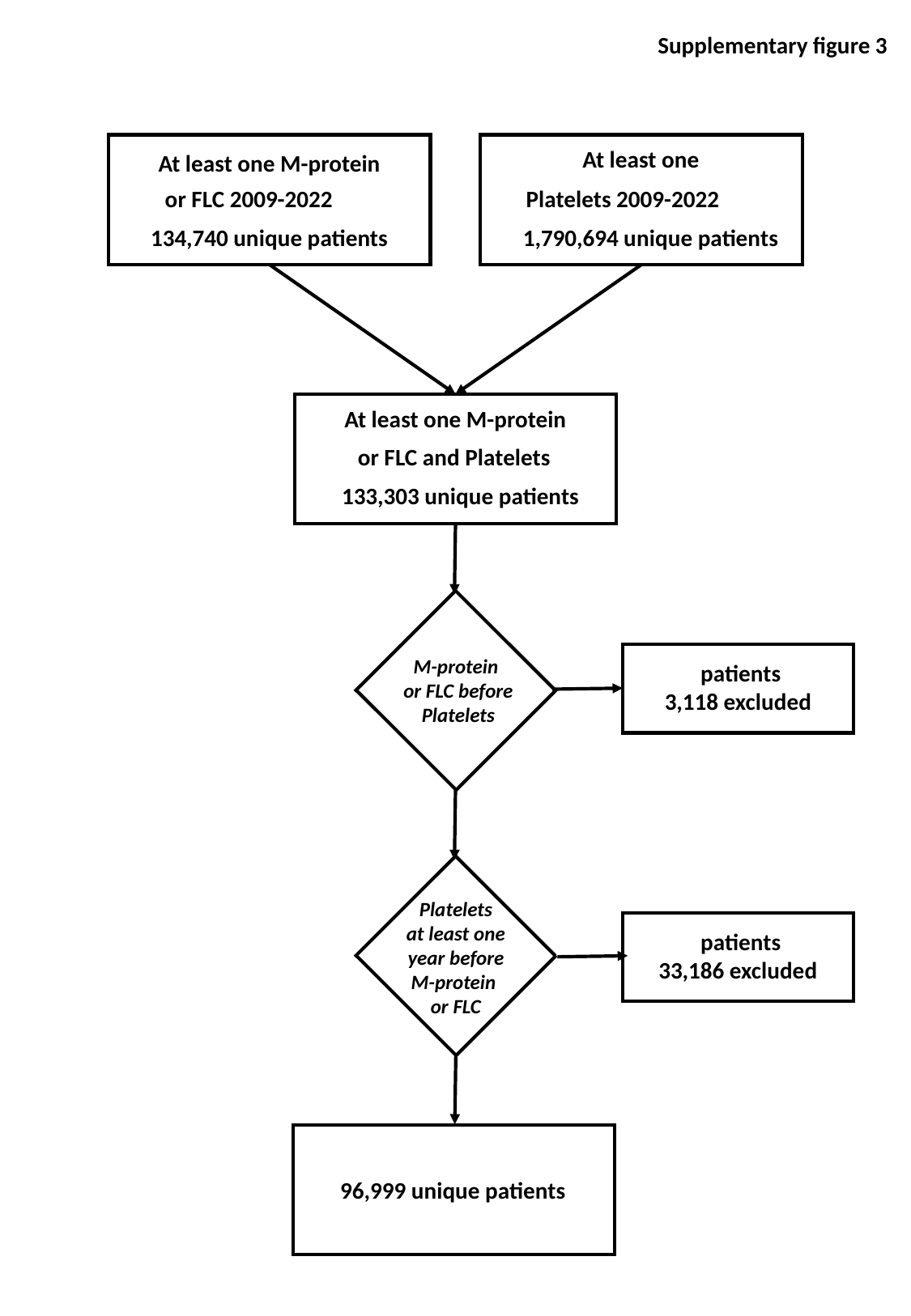

Supplementary figure 3
At least one M-protein
or FLC 2009-2022
134,740 unique patients
At least one
Platelets 2009-2022
 1,790,694 unique patients
At least one M-protein
or FLC and Platelets
 133,303 unique patients
M-protein
or FLC before
Platelets
 patients
3,118 excluded
Platelets
at least one
year before
M-protein
or FLC
 patients
33,186 excluded
 96,999 unique patients
